# A Smartphone App Designed to Empower Patients to Contribute Toward Safer Surgical Care: Qualitative Evaluation of Diverse Public and Patient Perceptions Using Focus Groups

**DOI:** 10.2196/24065

**Published:** 2021-04-08

**Authors:** Stephanie Russ, Nick Sevdalis, Josephine Ocloo

**Affiliations:** 1 Centre for Implementation Science King's College London London United Kingdom

**Keywords:** patient safety, mobile health, patient involvement, perioperative care, smartphone app, mobile phone

## Abstract

**Background:**

MySurgery is a smartphone app designed to empower patients and their caregivers to contribute toward safer surgical care by following practical advice to help reduce susceptibility to errors and complications.

**Objective:**

The aim of this study is to evaluate service users’ perceptions of MySurgery, including its perceived acceptability, the potential barriers and facilitators to accessing and using its content, and ideas about how to facilitate its effective implementation. The secondary aim is to analyze how the intended use of the app might differ for diverse patients, including seldom-heard groups.

**Methods:**

We implemented a diversity approach to recruit participants from a range of backgrounds with previous experience of surgery. We aimed to achieve representation from seldom-heard groups, including those from a Black, Asian, and minority ethnic (BAME) background; those with a disability; and those from the lesbian, gay, bisexual, transgender, queer (LGBT+) community. A total of 3 focus groups were conducted across a 2-month period, during which a semistructured protocol was followed to elicit a rich discussion around the app. The focus groups were audio recorded, and thematic analysis was carried out.

**Results:**

In total, 22 individuals participated in the focus groups. A total of 50% (n=11) of the participants were from a BAME background, 59% (n=13) had a disability, and 36% (n=8) were from the LGBT+ community. There was a strong degree of support for the MySurgery app. The majority of participants agreed that it was acceptable and appropriate in terms of content and usability, and that it would help to educate patients about how to become involved in improving safety. The *checklist-like* format was popular. There was rich discussion around the accessibility and inclusivity of MySurgery. Specific user groups were identified who might face barriers in accessing the app or acting on its advice, such as those with visual impairments or learning difficulties and those who preferred to take a more passive role (eg, some individuals because of their cultural background or personality type). The app could be improved by signposting further specialty-specific information and incorporating a calendar and notes section. With regard to implementation, it was agreed that use of the app should be signposted before the preoperative appointment and that training and education should be provided for clinicians to increase awareness and buy-in. Communication about the app should clarify its scientific basis in plain English and should stress that its use is optional.

**Conclusions:**

MySurgery was endorsed as a powerful tool for enhancing patient empowerment and facilitating the direct involvement of patients and their caregivers in maintaining patient safety. The diversity approach allowed for a better understanding of the needs of different population groups and highlighted opportunities for increasing accessibility and involvement in the app.

## Introduction

### Background

Health care is being called to better embrace the potential of digital technology for transforming patient care. The unprecedented spread of mobile digital technologies and their potential to address health priorities has evolved into the field of mobile health, which can be defined as “medical and public health practice supported by mobile devices” [1]. Smartphone apps, as an example of such technology, have emerged as a key device for communicating health information at scale and for improving patient empowerment, which is an important long-term objective of health care internationally [2-6]. A growing evidence base suggests that smartphone technology may play an important role in improving mental and physical health outcomes for a range of patient groups in both high- and low-income countries [7-11]. In the future, the use of smartphone technology to enable patients to be more involved in their care and to connect them with their providers outside the clinic is likely to be even more important. The recent COVID-19 pandemic offers a prime example of a health system response that has relied on the implementation of remote and largely digital solutions for delivering and receiving care.

**Figure 1:**
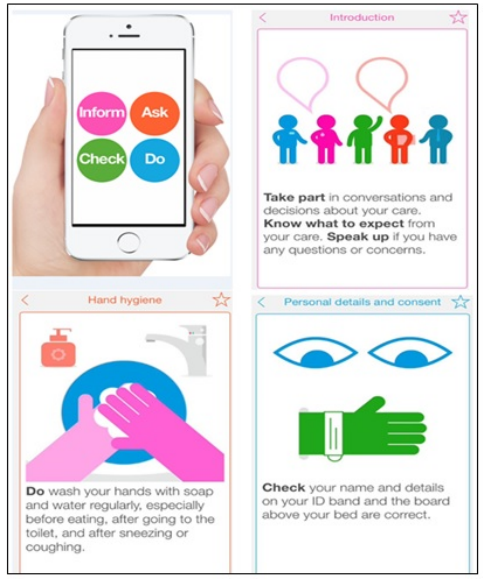
MySurgery app screenshots.

This paper focuses on MySurgery, an app for use within the context of surgical care. MySurgery is a smartphone app designed to empower patients and their caregivers to play a role in improving safety in surgical care in the National Health Service (NHS) of the United Kingdom. MySurgery was created by a multidisciplinary team of clinicians, patient safety experts, and patient and public representatives and is available for free download on Apple devices from the App Store; to date, it has had more than 6000 downloads [12]. The animated, jargon-free app is centered around key areas of evidence-based surgical risk, such as medication management, consent and identification, wound care, falls, and hand hygiene (Figure 1). The app is designed to be used as an optional supplementary tool, rather than replacing any existing surgical educational material or safety procedures. It provides practical step-by-step advice on the actions patients and their caregivers can take to help mitigate risks, including warning signs to look out for, things to do, information to provide, and questions to ask. The objective is to encourage behaviors that will help to reduce a patient’s susceptibility to error, for example, by flagging up inconsistencies or omissions, by encouraging behaviors that reduce the risk of infection or falls, and by providing information to allow better clinical decision making. It also helps patients to prepare optimally before and after surgery. Previous research has identified these as the kind of *safety-related behaviors* that should be incorporated into interventions designed to enhance patient involvement in safety [13-15]. The interventional rationale behind MySurgery is much in keeping with that of enhanced recovery programs, which are evidence-based perioperative programs that employ a multidisciplinary and multimodal approach based on implementing a checklist of actions that help resolve issues that delay recovery and cause complications [16]. These programs have been shown to significantly reduce morbidity, mortality, and length of hospital stay [17,18]. In a recent pilot evaluation of MySurgery with a diverse group of 42 surgical patients, the app received positive feedback [19].

The potential benefits of mobile apps such as MySurgery can only be realized if they are deemed acceptable by the end user and the initial intent to use the intervention translates to actual user engagement with it. Engagement with a digital intervention can be conceptualized as the extent (eg, amount, frequency, duration, and depth) of use and a subjective experience characterized by attention, interest, and affect [20]. Understanding the potential barriers to and facilitators of engagement before the implementation of an intervention is critical for successful implementation. A number of technology adoption models have been proposed that define the kind of factors that will influence perceived acceptability and intention to use digital interventions, ranging from perceived ease of use, social influences and subjective norms, requirement for access to personal data and related security concerns, perceived usefulness, and perceived behavioral control (or perceived ability to carry out a given behavior promoted by the app) [21-23]. The influence these factors have on engagement will vary based on different features of the app at hand (eg, how much personal data input an app requires); however, it is also likely to vary for different patient groups. For example, those from different cultures or with certain disabilities may experience different barriers to and facilitators of using the app. These potential group differences have rarely been explored in app usability and evaluation studies.

### Objectives

This study has 2 aims. The first aim is to explore in-depth views about MySurgery held by individuals who had previously undergone surgery, using focus groups. Focus groups are a fruitful way to gather the perceptions and experiences of groups of service users that offer them more control of the interaction [24]. Specifically, we aim to understand the perceptions related to the following:

1. The content and usability of MySurgery, including its perceived acceptability and usefulness
2. The perceived potential impact of using MySurgery on the doctor-patient relationship
3. Potential barriers to and facilitators of using MySurgery and associated strategies for overcoming any barriers
4. Potential strategies for introducing MySurgery into surgical care pathways in the UK NHS

The second aim is to implement a diversity and inclusion approach for recruitment, which would allow the research to address weaknesses in the literature to date. There are long-standing criticisms about tokenism and the lack of diversity in involving patients and the public in health care and health care research [25,26]. By targeting seldom-heard groups in the recruitment process, we aim to ensure that their representation will enable us to gather feedback from a broader spectrum of the population for whom the app is designed. We hypothesized that the views held regarding the app, specifically perceived barriers to using it in practice, would vary according to sociocultural factors and disability status. Therefore, we felt that a diversity approach was important.

## Methods

### Participants

To implement the diversity and inclusion approach, as described later, the recruitment of individuals for the focus groups took place via 2 routes. The first route was to advertise on *People in Research*, which is a UK-hosted website designed to provide opportunities for the public to become involved in NHS, public health, and social care research. A bespoke study advert (Multimedia Appendix 1) was published on the website, offering participation in the focus groups or involvement in the project steering group (the project steering group met biannually to oversee the wider research program and consisted of public and patient representatives, clinicians, and patient safety scientists. It is not described further here). Interested individuals could respond directly to the study team to express their interest in taking part. The second route was to email the project advert to individuals who had asked to be informed of upcoming research opportunities through the Patient and Public Involvement (PPI) Group at the Centre for Implementation Science (CIS), King’s College London, where the research was hosted. The criteria stipulated that individuals should only apply if they were over the age of 18 years and could speak, understand, and read English. We also set criteria that they should have experienced surgery within the past 5 years, such that they could draw on their experience to respond to the questions, which were focused on patient involvement in surgical care.

### Diversity and Inclusion Approach

As part of the study aims, we implemented a diversity and inclusion approach whereby we sought to include representation from individuals from seldom-heard groups. This approach included the following elements:

1. We used the Equality Act 2010 [27] to inform our conceptualization of diversity, which sets out 9 protected characteristics that may be the subject of discrimination. We chose to focus on 3 of these characteristics, which relate to groups that have traditionally been underrepresented in health care research: disability, race, and sexual orientation [28,29].
2. Using the study advert (Multimedia Appendix 1), we invited anyone with previous experience of surgery to participate in the project. However, we also set out *desired criteria* focused on the aforementioned 3 protected characteristics, encouraging the following groups in particular to apply: those with a disability; those from a Black, Asian, and minority ethnic (BAME) group; and those from the lesbian, gay, bisexual, transgender, and queer (LGBT+) community.
3. The CIS PPI Group (one of the aforementioned routes through which we recruited participants) had just been set up using a local community-based diversity approach, which had drawn upon the Equality Act. Therefore, it had a contacts list with a wide diversity of groups, which we felt would assist in the recruitment of a diverse sample.
4. We drew upon the cultural competence and expertise of JO as a researcher from a BAME background, with long-standing expertise working on diversity issues.
5. An *equality monitoring form* (Multimedia Appendix 2) was used to monitor the diversity of responses to our advert with sections of the form based on categories in the Equality Act 2010. We then aimed to ensure that we represented a diverse range of groups by selecting at least 50% of our sample from those stipulating that they held 1 of the 3 protected characteristics.
6. Before the commencement of the focus groups, the *rules for engagement* were discussed and agreed upon by all, with the aim of breaking down any potential barriers to inclusion. These included practical points such as turning off mobile phones and points on communication style, such as not speaking over one another, allowing everyone to speak, not needing permission to speak, and being able to respond directly to one another. Participants were reassured that there were no right or wrong answers and that we were not trying to reach a consensus but rather to gather information and understand a range of different viewpoints.
7. To encourage participation from diverse groups, all participants received a one-off payment of UK £40 (US $56), a lunch following the focus group, and their travel expenses.

### Design

Three 90-minute focus groups were conducted across a 3-month period (November 11, 2017, to January 1, 2018). Each focus group had a maximum capacity of 10 participants and was facilitated by 2 researchers with previous experience and training in facilitating focus groups (SR and JO). The project was reviewed and approved by the King’s College London Ethics Committee (REF: MR/17/18-108).

### Materials

#### Equality Monitoring Team

To measure the diversity of our sample, we asked participants to complete a standardized equality monitoring form (Multimedia Appendix 2), which captured demographic information, including age, ethnicity, disability status, and sexuality.

#### Bespoke Focus Group Questionnaire

To better understand our sample, we asked individuals to complete a short bespoke questionnaire (Multimedia Appendix 3) that captured information relating to their previous experience of having had surgery (including date of last surgery and how many procedures they had had in total) and their familiarity with using smartphone apps.

#### Semistructured Discussion Guide

A semistructured discussion guide was devised to assist the facilitators in guiding the conversation during the focus groups. It included questions and prompts structured around the study aims and a rough schedule to ensure everything was covered in the available time, building in room for refreshments and comfort breaks (Multimedia Appendix 4). Thinking about taking an inclusive approach was particularly important; this meant thinking about room access, options for any special diets, developing ground rules to ensure everyone was able to participate equally, and building specific questions to prompt discussion about how different groups might be able to use the app. For example: Will the app be acceptable to all?; For whom will the app be less useful?; What about people who do not own or use a smartphone or iPad?; How can we get around the issues identified?; Can you think of alternative ways of delivering this information?; and Should this impact on the decision to take the app forward?

### Procedure

Individuals who responded to the study advert were sent a more detailed study information sheet in the post or via email. Those who were still interested in being involved were sent the study questionnaires (equality monitoring form and bespoke focus group questionnaire) and asked to bring the completed forms to their focus group, which was booked for 1 of 3 dates in November or December 2017. If they had access to an Apple device, they were asked to download and familiarize themselves with the MySurgery app before attending their focus group.

On the day of the focus group, participants arrived 30 minutes before the commencement of the discussion to allow time to complete their informed consent, meet the other group members, and get refreshments. They could also use this time to familiarize themselves with the MySurgery app (on study iPads), if they had not been able to at home or if they wanted to refresh their memory. The focus group itself was preceded by a 10-minute presentation by the 2 facilitators, to introduce themselves and the aims of the study and to provide a brief background on the development and objectives of the MySurgery app. Following this, the participants introduced themselves, and the *rules for engagement* in the focus group discussion were outlined. Relevant ethical issues such as how data would be stored and used and the participants’ right to withdraw were also reviewed.

The discussions were audio recorded (with participants’ permission) for subsequent transcription for analysis purposes and lasted for 90 minutes, with a 10-minute comfort and refreshment break.

### Data Analysis

The audio recordings of the 3 focus groups were transcribed to allow a qualitative thematic analysis of the discussion. This was completed separately by 2 researchers (SR and JO) with expertise in qualitative data analysis. An inductive approach to the analysis was undertaken whereby the raw textual data were used to extract themes based on exploratory interpretation of the material, providing a summary of its content. This approach enables the development of a theory about the perceptions held by the focus group participants, with clear links to the research objectives [30]. Practically, this was achieved through the following steps:

1. Focus group transcripts were combined and rearranged to group answers together for each interview protocol question.
2. For each question, we noted the main ideas that were raised in the discussion.
3. We reviewed the main ideas (across all questions) to identify ideas that were raised again and again or engendered a particularly rich discussion.
4. The researchers performed critical thinking about these recurring ideas to identify emergent themes.
5. We identified quotations from the transcripts to illustrate each theme.

To establish coding agreement between the researchers, initially half of the transcripts were analyzed by both researchers, and the extracted themes were compared and agreed upon. Subsequently, the remaining half of the transcripts were divided equally, and the researchers came together at the end to agree on the final emergent themes.

## Results

### Participants

A total of 22 individuals participated in the focus groups (group 1: n=8, 36%; group 2: n=9, 41%; and group 3: n=5, 23%). The participant characteristics are summarized in Table 1. There was an even spread of males and females, and the groups were diverse according to age, ethnicity, disability, and caregiver status. Just over one-third (n=8, 36%) of the sample was from the LGBT+ community. All participants had undergone surgery within the past 5 years, excluding one individual who was awaiting upcoming surgery.

**Table 1:** Participant characteristics (N=22).

Characteristic			Participants, n (%)
**Sex**			
	Male		10 (45)
	Female		12 (54)
**Age (years)**			
	18-24		1 (5)
	25-34		2 (9)
	35-44		2 (9)
	45-54		4 (19)
	55-65		9 (43)
	>65		3 (14)
**Ethnicity**			
	White		11 (50)
	**BAME^a^**		11 (50)
		Asian/Asian British	3 (14)
		Black/African/Caribbean/Black British	5 (23)
		Mixed/multiple ethnic groups	3 (14)
**Sexual orientation**			
	Heterosexual		14 (64)
	Gay		3 (14)
	Lesbian		1 (4)
	Other		4 (18)
**Disability**			
	Yes		13 (59)
	No		9 (41)
**Caregivers**			
	Yes		9 (41)
	No		13 (59)
**Number of previous surgeries**			
	0		1 (4.5)
	1-2		9 (41)
	3-4		10 (45)
	≥5		2 (9)
**Smartphone user**			
	Yes		20 (91)
	No		2 (9)
**Would you use a smartphone for health-related purposes**			
	Yes		19 (86)
	No		3 (14)

^a^BAME: Black, Asian, and minority ethnic.

### Thematic Analysis of Focus Group Discussions

The following themes were extracted from the focus group transcripts. Illustrative quotes are provided in Multimedia Appendix 5.

#### Perceptions Regarding Patient Involvement in Safety

There was a strong sense of support for the concept that patients should be closely involved in the safety of their own care. Some participants were very confident about this notion:

Nothing about us, without us.

It’s about taking responsibility for your care and we need to be moving in that direction.

A number of areas were highlighted where most patients do naturally become involved in the safety of their care, including complying with instructions around food, drink, and medications; attending preoperative appointments; and asking questions. However, it was suggested that there are areas where it might be difficult to become involved in safety, particularly where it involves conflict behaviors such as asking many questions, challenging the decisions of health care workers, or highlighting inconsistencies, suggesting that this may have a negative impact on the doctor--patient relationship and erode trust. For example, in some cultures, it is frowned upon to question a figure of authority (it was suggested that some minority groups might feel that such behaviors might affect the standard of care they receive), those with less assertive personality types might simply feel unable to engage in these behaviors, and others may feel more comfortable with the passivity of the traditional patient role:

What if you’ve got a patient who actively just wants to be told what to do, doesn’t want to have that responsibility.

It was agreed that for these areas particularly, facilitation to become involved in safety would be needed, including encouragement and empowerment from the health care workers themselves, information around how to contribute, and education around the broader safety-related areas patients and their advocates can feasibly influence:

Patients can get involved in patient safety but they need encouragement by clinicians and to be told in what ways they can contribute.

The importance of the involvement of family, friends, and caregivers was also highlighted, particularly for individuals who were too unwell to be involved themselves.

#### MySurgery App: Concept, Content, and Usability

MySurgery was endorsed as a positive step toward patient empowerment and an acceptable approach to facilitating patient involvement in safety. In a practical sense, the app was recognized to help participants prepare before and after surgery, for example, by detailing what to take into hospital and how to care for surgical wounds. From a safety perspective, participants agreed that by enhancing their knowledge of surgical risks and listing specific actions for mitigating them, the app shows the possibility of becoming more involved in safety and very real opportunities for avoiding errors or complications, regardless of the type of procedure being performed:

I think they (patients) ought to know what they can and can’t do, and if they can ask questions and it’s a two-way communication. That’s what this app seems to do, it shows people the possibilities, shows patients the possibilities.

It was agreed that MySurgery raises safety matters that might be of relevance but that might not have been thought about and that patients could address these issues using their own approach and style. In particular, MySurgery was recognized as a useful communication tool, informing patients about the areas they may wish to discuss with their health care team and about the information they should provide:

It just empowers you in a way because it doesn’t mean you have the confidence to say, have you washed your hands, but maybe next time you’ll be able to or maybe you can attempt to, or know that that’s important, or discuss it in a different way with the health worker.

It was stressed, however, that unless users of the app understood the context of patient empowerment and involvement within which the app sits, they might not fully grasp its relevance and importance, which may prevent buy-ins.

In terms of detail, participants liked the level of content in MySurgery in that it was *not information overload* and was clean, clear, and simple, with no jargon, even for individuals who struggle with medical information. The *checklist-like* layout of the app content was approved, as participants liked the feeling that they could cross items off their list and that they had *covered off all of the important things*. As a quick aide-memoire, the *top 10 things to remember* tab within the app was also popular. The app content was deemed generic enough to be appropriate for all surgical patients; however, it was discussed that those having very minor procedures or multiple procedures may only need to check the *top 10* section or may only be interested in 1 or 2 sections. In terms of usability, the app was described generally as being user friendly, easy to navigate, and nicely animated:

I liked it, I thought it was very user friendly, it wasn’t information overload so when you opened it and thought, oh god, I’m going to have to sit and spend ages reading through loads of...but it’s quite interactive and it’s short.

Some suggestions were made to improve MySurgery. Currently, the app requires you to work through it in a set order, meaning that later sections cannot be accessed until earlier sections have been completed. This was not a popular feature, as participants felt that they wanted to access whichever section looked most relevant to them, skipping other sections (eg, those having repeat or very minor procedures may only want to look at 1 or 2 sections). It was widely agreed that this limitation should be addressed. Others suggested that although the level of content was good, some users would like more details and information, perhaps specific to the procedure they are having done, and links and signposts to this kind of information could be built into the app:

How about links to information about the procedure you are having, or somewhere to find more information?

Some further suggestions were made around personalizing the app and incorporating more general support for managing one’s care, including the addition of a calendar to provide alerts for appointments or medications, a contacts list to record names of key clinicians, and an area in which to make notes. Finally, it was suggested that if you could enter your surgery date into the app, alerts could then be provided to remind you to check it. The suggested improvements to MySurgery are summarized in Textbox 1.

**Summary of findings: recommendations for the development and implementation of MySurgery.**

Content

- Include links and signposts to further resources and specialty-specific content.
- Include a calendar for inputting appointments with an integrated alert system, for example, “check you have everything before attending hospital tomorrow.”
- Include an area to make notes, for example, to record clinicians’ names and important phone numbers.
- App store information—make it clear that the use of the app is optional and noninterventional (ie, education only) and communicate the objectives of the app in plain English.

Usability

- Remove the requirement to work through the app in a set order.

Accessibility

- Develop audio and easy-to-read versions of the app.
- Make MySurgery available for Android devices.
- Distil the information into appropriate format for hard copies of the app, for example, booklet, leaflet, or posters.
- Make MySurgery available in different languages.
- Include information around where to find help in using the app.

Implementation strategy

- MySurgery should be recommended to patients before their preoperative appointment.
- Promote the use of the app through inclusion in the preop letter, via posters in primary care General Practice surgeries and pharmacies, and by displaying on the television screens in hospital wards.
- Educate clinicians to secure buy-in and promote the use of the app.
- Involve clinical teams and patients in adapting the app to specific surgical specialties or hospital units.
- Include information around how to download the app.
- Include guidance on how to use the app in practice.

#### Accessibility

The degree to which MySurgery is accessible and inclusive was an important point of discussion. If a safety intervention is accessible to some but not others, there is a moral issue to be considered in terms of equity, with some being unable to access information that might enhance their care. The viewpoints around this were varied, and the discussion was rich. MySurgery is currently available on Apple devices, meaning that those who cannot access an Apple device will not be able to benefit from the information within the app. Examples of groups falling into this category are those with Android devices, those who do not have access to a smartphone or tablet, those who find apps and technology difficult to use, those who do not speak English, those with visual impairments, those with learning difficulties or dementia, and those within institutions:

I think that the app is very clear and the language is very clear however there might be groups of society who have a lot of surgery that find it difficult. I’m talking about learning disabilities, Alzheimer’s and so on..., or people that have had neurological trauma and also people who might already be very unwell in hospital.

It is very important to consider how the accessibility of technology to these diverse groups can be enabled or improved. For example, MySurgery will soon be available on Android devices. Other adaptations might include developing an audio version of the app, developing easy-to-read versions, translating the app into different languages (so far, the app is available in English and French), developing paper-based versions, and considering whether the app needs to be adapted for situations where it will be used by a caregiver as opposed to the patient themselves (eg, when the patient is a child, has dementia, or is too sick to use the technology themselves).

The use of MySurgery by older individuals (ie, 65+ years) was also discussed, as there was a question around how accessible smartphone technology is to this population. There was widespread agreement that although certain individuals in this bracket may not be as familiar with smartphone technology as others, older individuals in general are increasingly comfortable with technology and are equally likely to own and use a smartphone or tablet (this is supported by the national survey data) [31]. Thus, it should not be assumed that older individuals will struggle to access the intervention; however, alternative formats, such as a hard copy of the app content, might be preferred:

Don’t make assumptions about the elderly...[be]cause there is a presumption that the elderly don’t like technology. I’ve spoken to some people in their 70s, I never say to them, do you know how to use an iPad, you just hand it over.

Another group of individuals were identified who have access to technology but would not wish to engage with this kind of information or do not want to know about risk—the traditionally passive patient described earlier. This reinforces the point that the use of technology should not be forced but rather presented as an option for enhancing care.

As a positive endorsement of MySurgery, there was agreement among most members of the group that the identified barriers to accessibility, although important to address, should not prevent investment in or implementation of the app, particularly where substantial improvements in the efficiency and safety of care might be realized by its use for so many:

You cannot with one thing reach all people. You probably won’t be able to reach a certain percentage, but the point is it’s actually much more efficient for most.

The group agreed, therefore, that as an intervention, MySurgery should be taken forward but with the support of efforts to make adaptations to improve adoption and accessibility where necessary.

#### Implementation

Participants agreed on some key factors that would be important to the implementation success of MySurgery (see Textbox 1 for a summary of these findings). First is the adoption of a multifaceted approach that targets patients before their preoperative appointment. The preoperative appointment typically takes place within a few weeks before surgery and is an opportunity for the patient (who may attend with a relative or friend) to discuss their upcoming surgery and to ask questions. Viewing the app before the appointment would mean they would have time to digest the information and to prepare any questions or information they feel are relevant, also giving them time to take any necessary actions before their procedure. A number of suggestions for signposting patients toward the app at this time point were made. These included mentioning the app in the preoperative letter (or including a flyer with the letter) with information about it and instructions about how to download it; providing information about the app on posters or on-screen adverts in pharmacies, GP surgeries, and hospital waiting rooms; or having tablets available in waiting rooms for patients to explore the app:

The letter that goes out to you says, you might want to look at this app first of all and if you have any worries about patient safety, bring them up in the interview, in the consultation. That is embedded in part of the consultation process and accepted.

The app could also be made available in paper version for those who find it difficult to use the technology. It will be important to make use of the NHS digital champions linked to trusts to help support implementation and to aid with adaptations such as this that might make it more accessible.

The second factor raised by the participants was that the implementation process should educate not only patients about the app but also clinicians. Fundamentally, MySurgery, like any safety intervention, should be something that all stakeholders, including clinical staff, are aware of and bought into to optimize its effectiveness. In the case of MySurgery, having buy-in and awareness from the clinicians would mean they could recommend the app if the patient had not yet heard about it, promoting uptake and endorsing its validity, and in doing so encourage patients to engage in safety-related behaviors. This sends the signal to the patient that their involvement is considered appropriate, as opposed to feeling that they are challenging or questioning the clinician’s ability. It would also help to reduce any potential strain on the patient--doctor relationship resulting from a patient mentioning an intervention the clinician is unaware of:

There’s a dual process going on here. As well as education of patients and empowering them, it’s also the education of the clinicians and I think it’s imperative that you speak to clinicians about it, so the onus isn’t on you to have to bring it up.

The next point around implementation focused on the content of the communications put out around the app. One potential untoward effect identified during the discussion was that the nature of the app content, which is focused on patient behaviors that can mitigate risks to safety, may be uncomfortable or even anxiety provoking for some patients. A second potential issue was that patients may feel that they *have* to use the app but may potentially struggle with accessing it or using the technology. To address these concerns, it was agreed that several points should be set out clearly in any promotional material around the app, including preoperative letters, poster or flyers, verbal communications, the App Store description of the app, and the introductory content of the app itself. It should be made clear that use of the app is supported by the NHS but that it is optional and supplementary to the safety procedures already in place. The context and scientific basis of the app (including the context of patient empowerment in which it sits) should be clarified in plain jargon-free English such that users understand why the use of the app may be beneficial and precisely what the app is trying to achieve. It should be made clear how to download the app, how to access help in using the app, and how the app content can be accessed if the users do not have a smartphone or tablet. Finally, examples of how to use the app in practice should be set out to give users ideas about where and how they might apply it to their care:

If you want some help to access this app or would like to discuss the information, you could contact your local A, B, C...

Finally, there was some conversation around creating specialty-specific versions of MySurgery, which are tailored to the specific risk profiles of different surgical specialties with more specific information around procedures and related recovery advice. There was also the suggestion of tailoring the app for specific hospital departments or units and integrating it with existing protocols and procedures to engender buy-in from staff and to help streamlining.

## Discussion

### Principal Findings

MySurgery is a smartphone app designed to empower patients and their caregivers to contribute toward safer surgical care. The animated checklist-type tool educates patients about simple behaviors they can undertake to mitigate a number of key evidence-based risks that are relevant to any hospital-based surgical procedure, with the objective of preventing avoidable surgical and medical complications. In this study, we conducted focus groups with 22 diverse service users who had experience of surgery to explore in-depth perceptions about the acceptability of the app, approaches to aid effective implementation, and strategies to address barriers to inclusion and accessibility.

As a general concept, participants supported the idea of patient involvement in safety but felt that education around *how* to become involved was important and that some would need assistance in doing so, which is in line with previous research [32]. MySurgery was endorsed as an acceptable approach to facilitating patients to become involved in safety by setting out key areas of risk that patients are able to influence (but which they may not have been aware of) and the safety-related behaviors they can participate in to mitigate their risk. The app was deemed acceptable in terms of content and usability, and participants agreed that by becoming better informed and understanding where problems might arise, use of the app should assist communication with health care professionals. It was also deemed probable that use of the app would reduce susceptibility to avoidable errors, given that patients would be more likely to capture errors themselves or would be more active in helping to avoid error-inducing conditions by following the advice. In this sense, patients would act as an extra safeguard, adding to the available resources in the health care system for improving safety. Some important suggestions for improvement to the app were provided, both in terms of content (eg, including links to more detailed procedure-specific information) and usability (eg, making it possible for users to access all sections of the app rather than having to work through it in a specified order).

The study also offered important insights into the diversity and inclusivity of its end users in terms of how to make the app accessible to as many patients as possible. By virtue of the recruitment approach undertaken, we achieved a diverse sample, including representation from seldom-heard groups such as those with a disability and those from a BAME background. This contributed toward a rich and varied conversation with reflections around the acceptability of MySurgery and barriers to its use from many different perspectives. There were some examples in which the app was deemed to be well set up in terms of accessibility, such as the level of detail, animation, and avoidance of jargon, making it accessible to those who might not be comfortable or familiar with medical information. However, the discussion also identified areas where further development is required to make the app accessible to certain groups or where barriers to using the information in the app may arise.

Three key groups of individuals were identified who may experience different kinds of barriers to using MySurgery. The first were those who would like to use the app but cannot, for very practical reasons, access it in its current format, for example, those with visual impairments, those with learning difficulties, those who do not have access to a smartphone or tablet or who find technology difficult to use, those in prison or other institutions, and those who do not speak English. Various adaptations to the content and presentation of MySurgery have been suggested to address the barriers to inclusion, which should be explored and built into the ongoing development of the app. The issue of *digital exclusion* has been illuminated during the recent COVID-19 pandemic, which has seen those in certain groups, such as those who cannot afford the data required to download and interact with apps, disproportionately set back in a number of respects [33]. Alternative approaches to accessing the content of apps, such as MySurgery, must be made available to such groups.

The second group identified were those who would like to use the app but who felt they would encounter difficulties in acting on the information and advice provided. This may be due to *intrapersonal barriers* (ie, relating to the user of the app themselves), for example, if they are shy or feel psychologically vulnerable in some way, which means they find it difficult to become involved; interpersonal barriers (ie, between the user of the app and health care professionals), for example, where patients or professionals struggle to express themselves in a way that is easy to understand; or *cultural barriers (on behalf of the user of the app and/or the health care professional)*, for example, where some patients from certain cultures might deem it inappropriate to question a person of authority or if health care professionals are resistant to patient involvement. The extent to which the barriers impede patient involvement may vary from behavior to behavior. For example, some behaviors recommended by the app can be completed privately with very little interaction with health care workers, for example, ensuring nonslip footwear is worn and caring appropriately for surgical wounds following discharge, whereas other behaviors rely heavily on effective communication and may be more strongly influenced by the barriers described, for example, providing information about medicines and medical history or checking if health care workers have washed their hands. Previous work on patient involvement in safety predicts that these kinds of barriers will arise and outlines the importance of overcoming them by understanding alternative approaches to empowering patients [13]. This will require ongoing work with these patients to understand which approaches to empowerment would be acceptable to them. A final group was identified who may not wish to use the app at all, for example, because they find it anxiety provoking or because they prefer to take a more passive role. For these individuals, it should be made clear that use of the app is optional. However, further work should also explore how to repackage the content of MySurgery and other such interventions into a format that may empower and educate these individuals without pushing them outside of their comfort zone or placing a perceived additional burden on them at a time when they may already feel anxious. When patients become better informed and prepared for their surgery (eg, by being exposed to the information in the app), safety-related behaviors such as communication with health care workers should improve naturally [15].

These findings highlight an important point for app developers in the health care sector. Although there are clearly many benefits of apps in mobilizing knowledge, it is naïve to think that apps alone democratize access to information. As demonstrated here, there are cohorts of the population who, for a range of reasons, are unable or unwilling to use health apps, even when they have access to the technology. These barriers to use may only be revealed when examining closely the perceptions of users from a diverse range of backgrounds. It may not be possible to overcome every barrier to app use, and it may be that certain apps remain unsuitable for certain individuals. However, in this study, given the broad support for the MySurgery app, it was agreed by the participants that recognizing the presence of barriers to use should not prevent its development or future implementation but rather be used to enhance the implementation process by building strategies to improve accessibility. One approach that is likely to be important for encouraging use across all patient groups is the provision of support and encouragement for patient involvement from health care professionals themselves, particularly for those behaviors that patients perceive as potentially confrontational (eg, asking a health care professional if they have washed their hands before examining them). Indeed, the important role of health care professionals in promoting the use of MySurgery was a strong emergent theme in this study and is a well-established finding in the safety literature [32,34].

Once the suggested improvements have been made to MySurgery, the next step will be to trial implementation of the app on a small scale within 1 or 2 surgical departments. However, such interventions need to be initiated at the right time with the right tools to be effective; therefore, careful planning of the implementation strategy will be key. Several important points were raised in the discussions that should be fed into this approach. Regarding timing, it was agreed that users should be signposted toward the app just before their preoperative appointment when the information contained is most salient to them and when they have time to act on the advice, for example, by discussing safety-related matters with their clinician at their appointment and by ensuring they have followed the advice about preparing for surgery. It was also deemed critical to engage clinicians in the roll out of the app such that they are aware of the intervention and can promote it with their patients and empower them to discuss any concerns. This will likely involve not only education sessions and the appointment of clinical champions but also, as mentioned before, a long-term cultural effort to break down resistance to patient involvement. Finally, part of the implementation approach should focus on thorough communication of the remit and scope of MySurgery, the broader safety context within which it sits, and available assistance for downloading and using the app.

### Strengths and Limitations

Common to qualitative research of this type, we had a small sample size, which limits the generalizability of the results. However, the objective of choosing focus groups over quantitative approaches is that it allows for the generation of far-richer data sets, which are desirable during the early phases of evaluating an intervention and planning its implementation. We achieved data saturation in the analysis, which allowed us to understand in depth the perceptions of the intervention and a theory of how it might work and the various themes we discussed relating to its accessibility and implementation. By carefully planning a diversity approach, we had representation from individuals from groups that tend to be underrepresented in health care research, including those with a disability, those from a BAME background, and those from the LGBT+ community. We feel this enriched the data in making it more representative of the population, encapsulating wide-ranging views of individuals from diverse backgrounds. This resulted in several points being raised, particularly around the inclusivity of the MySurgery intervention, which may not have been captured otherwise. To achieve this diversity, it was important to plan the focus group meetings carefully to ensure that they were accessible to all, that financial compensation for time and travel was provided, and that all dietary requirements were catered for. Incorporating a diversity approach in research such as this will allow us to have a more nuanced way of understanding the needs of different population groups and therefore in addressing if and where digital exclusions apply and how they can best be addressed. Given the close links between engagement with a digital intervention and adherence to its content, establishing an understanding of the factors that will influence engagement is a critical activity that should be undertaken with direct input from service users early on in the process of app design. This helps in identifying the design features that will draw users to (or deter them from) the intervention in the first place and features of the app that will enhance user motivation and autonomy and personal relevance and credibility of the intervention for different groups [20,35].

### Next Steps

Going forward with this program of research, it will be important to triangulate our findings with the implementation science literature to provide a theoretical lens and systematic approach to finalize the implementation plan. Implementation scientists are interested in understanding how best to promote the uptake of research findings into routine health care, calling on theoretical approaches to provide better understanding and explain how and why implementation succeeds or fails. Applying implementation science theory can help to map out the entire implementation approach—using a single taxonomy to identify the factors that might influence implementation effectiveness, including identifying the relevant stakeholders, the likely barriers to and facilitators of implementation, and how these interact within the context at hand. If used to evaluate the initial stages of implementation, theoretical frameworks can help to produce findings to inform stakeholders on improvements to the intervention and its implementation. The literature also provides guidance in the selection of implementation strategies (ie, discrete methods or techniques used to enhance the adoption and sustainability of an intervention). Recent research has identified more than 70 discrete implementation strategies relevant to health care researchers, ranging from assessing readiness for change within an organization; providing supervision and training; tailoring the intervention to the specific context, right through planning marketing approaches; and considering incentive plans [36]. By consulting and cross-referencing these with the strategies identified as being important in this study and matching them to the barriers and facilitators identified, the approach to implementation becomes more robust, and strategies that may be helpful but may not have been considered can be built-in. In addition, by applying a theoretical framework to the evaluation of the implementation, it can aid in the implementation, which increases the efficacy of research and allows results to be more reliably generalized and built upon across future studies and contexts [37,38]. A later step will be to consider economic evaluation of the intervention to establish its role (if any) in cost saving. Such evaluation is rarely attempted, despite offering a more evidence-based assessment of the scalability, sustainability, and benefits of broader investment in such technology tools [39,40]. Finally, as raised in the focus group discussion, it seems likely that there will be interest from providers in tailoring apps such as MySurgery to specific specialties or hospital units, to achieve better streamlining and integration of processes of care. Adaptation of interventions to specific contexts in this sense is an important principle of quality improvement work of this kind, not least to engender increased buy-in from staff. We will, therefore, be looking to collaborate with NHS Trusts to produce tailored versions of MySurgery for evaluation going forward. We will continue to build on the theory and practice of incorporating a diversity and inclusion approach into this study, as we believe, for reasons already mentioned, that adopting this approach could have significant benefits in making interventions more effective.

### Conclusions

This study was successful in establishing a diverse and inclusive stakeholder group to provide formative in-depth feedback on the MySurgery app. The app was received enthusiastically. It was endorsed as a powerful tool for enhancing patient empowerment in general and an appropriate approach to addressing well-established aims to involve patients and their relatives directly in maintaining patient safety. Various adaptations to the app should be made to make it more accessible to certain groups, which will involve the development of a comprehensive and multipronged implementation approach informed by diverse stakeholders.

## Appendix

Multimedia Appendix 1 Study advert.

Multimedia Appendix 2 Equality monitoring form.

Multimedia Appendix 3 Questionnaire regarding previous experience of surgery and smartphone apps.

Multimedia Appendix 4 Focus group discussion guide.

Multimedia Appendix 5 Illustrative quotes.

## Abbreviations

BAME: Black, Asian, and minority ethnic
CIS: Centre for Implementation Science
LGBT+: lesbian, gay, bisexual, transgender, and queer
NHS: National Health Service
NIHR: National Institute for Health Research
PPI: Patient and Public Involvement
