# Supplementary material for: A Smartphone App Designed to Empower Patients to Contribute Toward Safer Surgical Care: Qualitative Evaluation of Diverse Public and Patient Perceptions Using Focus Groups
Source: JMIR Mhealth Uhealth. 2021 Apr 8;9(4):e24065. doi: 10.2196/24065 (PMC8063097; doi:10.2196/24065)
Supplement: Multimedia Appendix 1 [file mhealth_v9i4e24065_app1.docx]

**
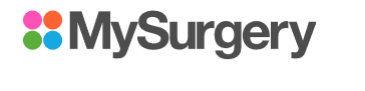

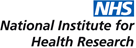
**
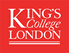


**Seeking: NHS Service User Participation in Focus Group**

**Research area: Safety in Surgery**

**Background**

Research suggests that around 1 in 10 patients entering NHS hospitals will experience an adverse event (an unintended error) in their care. As such, patient safety is a major priority for NHS England. Being at the centre of the process of care, patients and their families can help NHS staff to ensure their safety and improve their outcomes if they are appropriately informed of the potential risks.

**Project aims**

The aim of this research project is to understand patients’ views regarding a new smartphone app, called MySurgery (free to download on the App Store), which informs patients and their advocates about the key risks they face as part of having a surgical procedure and the actions they can take to improve their safety, outcomes and experience.

**How can you help?**

- **Current opportunity:** We are inviting service users to attend a focus group at King’s College London, Denmark Hill on the following dates…. The focus group will last approximately 2 hours and will consist of around 10 participants, facilitated by two researchers. You will be able to trial the MySurgery app and will be asked what you think of it in a number of different respects, including how you believe it could be trialled with surgical patients. The focus group will also discuss the design of the study moving forward and the content of future study materials (e.g. questionnaires).
- **Essential Criteria***:* You must have had surgery in the past 5 years OR be awaiting upcoming surgery. However, you do not need to have any ‘expert’ knowledge – we are purely interested in your views as a previous or upcoming surgical patient.
- **Desired criteria**: We are particularly interested in talking to groups who are seldom heard, for example those with a disability, those from a minority ethnic group and those from the LGBT+ community. However, everyone is encouraged to respond to this invitation regardless of their background.

**Remuneration**

You will receive a one-off payment of £40 for attending a focus group. You will also receive travel expenses and carer fees (e.g. child care). Over-night accommodation cannot be provided.

**[
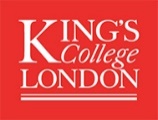
](https://www.google.co.uk/url?sa=i&rct=j&q=&esrc=s&source=images&cd=&cad=rja&uact=8&ved=0ahUKEwiFgdS9gevVAhVMJVAKHRkpBucQjRwIBw&url=https://www.kcl.ac.uk/study/postgraduate/index.aspx&psig=AFQjCNG_TcMArl0SVff410AmXRJu5A9UNg&ust=1503497148032211)Contact Information**

If you are interested in becoming involved in either a focus group or the steering group meetings please contact the lead investigator, Dr Stephanie Russ. You will be provided with further details at this stage.

Email: [stephanie.russ@kcl.ac.uk](mailto:stephanie.russ@kcl.ac.uk), Telephone: 0207 848 0663
