# Supplementary material for: A Smartphone App Designed to Empower Patients to Contribute Toward Safer Surgical Care: Qualitative Evaluation of Diverse Public and Patient Perceptions Using Focus Groups
Source: JMIR Mhealth Uhealth. 2021 Apr 8;9(4):e24065. doi: 10.2196/24065 (PMC8063097; doi:10.2196/24065)
Supplement: Multimedia Appendix 2 [file mhealth_v9i4e24065_app2.docx]

#

# Equality Monitoring Form

## Why we are asking for this information

King’s College London is committed to promoting equality of opportunity, to ensure that everyone has the chance to participate fully in the research it supports.

In completing this form, you will help us understand who we are reaching and how to better include everyone in our community. The information will be used to provide an overall profile analysis of our Patient and Public Voice participants, and to make sure that we are fulfilling our commitments. You do have a right not to disclose the information; however, by doing so you may impact our ability to ensure equality of opportunity.

## Data protection

The information you provide is anonymous and will not be stored with any identifying information about you. We may use anonymized statistics and data you have provided to have discussions about how to improve the diversity and spread of our research and also in research reports and publications, but no information will be used in any way which allows any individual to be identified. All details are held in accordance with the Data Protection Act 1998.

The information that we need, as outlined in the 2010 Equality Act, includes information about age, disability, gender reassignment, marital status, maternity, race, religious belief, sex, and sexual orientation.

If you would like the form in an alternative format, or would like help in completing the form, please contact Dr Stephanie Russ: [stephanie.russ@kcl.ac.uk](mailto:stephanie.russ@kcl.ac.uk), 07725560505.

## Please select the boxes which are relevant to you

**Ethnicity**

Please select what you consider your ethnic origin to be. Ethnicity is distinct from nationality.

| **Asian/Asian British** | **Black/African/Caribbean/ Black British** | | | **Other ethnic group** | | |
| --- | --- | --- | --- | --- | --- | --- |
| Indian  Pakistani  Bangladeshi  Chinese  Any other Asian background | Caribbean  African  Any other Black/African/Caribbean background | | | Arab  Any other ethnic group | | |
| **Mixed/multiple ethnic groups** | **White** | | | **Rather not say** | | |
| White and Black Caribbean  White and Black African  White and Asian  Any other mixed/multiple ethnic background | English  Northern Irish  Scottish  Welsh  British  Irish  Gypsy/Irish traveller  Any other White background | | | Rather not say | | |
| **Age** | |  | **Sex** | | **Sexual orientation** |  |
| 10 - 14 | |  | Male (M) | | Heterosexual |  |
| 15 - 19 | |  | Female (F) | | Gay man |  |
| 20 - 24 | |  |  | | Lesbian |  |
| 25 - 34 | |  | Rather not say | | Other |  |
| 35 - 44 | |  |  | | Rather not say |  |
| 45 - 54 | |  |  | |  |  |
| 55 - 64 | |  |  | |  |  |
| 65+ | |  |  | |  |  |
| Rather not say | |  |  | |  |  |
|  | |  |  | |  |  |

**Gender re-assignment**

Have you gone through any part of a process (including thoughts or actions) to change from the sex you were described as at birth to the gender you identify with, or do you intend to? (This could include changing your name, wearing different clothes taking hormones or having any gender reassignment surgery).

Yes

No

Rather not say

| **Religion / belief** |
| --- |

| No religion |
| --- |
| Buddhist |
| Christian |
| Hindu |
| Jewish |
| Muslim |
| Sikh |
| Atheist |
| Any other religion |
| Rather not say |

| **Disability**  The Disability Discrimination Act 1995 (DDA) defines a person as disabled if they have a physical or mental impairment which has a substantial and long term (i.e. has lasted or is expected to last at least 12 months) adverse effect on ones ability to carry out normal day-to-day activities. This definition includes conditions such as cancer, HIV, mental illness and learning disabilities.  Do you consider yourself to have a disability according to the above definition? | | | |
| --- | --- | --- | --- |
| Yes, limited a lot | Yes, limited a little | No | Rather not say |
| If you selected yes, please indicate your disability: | | | |
| Vision (e.g. blindness or partial sight) | | | |
| Hearing (e.g. deafness or partial hearing) | | | |
| Mobility (e.g. difficulty walking short distances, climbing stairs, lifting and carrying) | | | |
| Learning, concentrating or remembering | | | |
| Mental health | | | |
| Stamina or breathing difficulty | | | |
| Social or behavioural issues (e.g. neuro diverse conditions such as Autism, Attention Deficit Disorder or Asperger’s Syndrome) | | | |
| Other impairment | | | |
| Prefer not to say | | | |

| **Carer responsibility**  Do you look after, or give any help or support to family members, friends, neighbours or others because of either:   - Long-term physical or mental ill-health / disability - Problems related to old age   Yes  No  Rather not say |
| --- |
| If you selected yes, please indicate your caring responsibility (select all that apply) |
| Primary carer of a child/children (under 18) |
| Primary carer of disabled child/children |
| Primary carer of disabled adult (18 and over) |
| Primary carer of older person (65+) |
| Secondary carer |
| Rather not say |

**If there is anything further you would like us to know, please provide details below:**
