# Supplementary material for: A Smartphone App Designed to Empower Patients to Contribute Toward Safer Surgical Care: Qualitative Evaluation of Diverse Public and Patient Perceptions Using Focus Groups
Source: JMIR Mhealth Uhealth. 2021 Apr 8;9(4):e24065. doi: 10.2196/24065 (PMC8063097; doi:10.2196/24065)
Supplement: Multimedia Appendix 3 [file mhealth_v9i4e24065_app3.docx]

**Focus Group Questionnaire**

The following questions are designed to capture your previous or upcoming experience of having had a surgical procedure, and also your familiarity with smartphone technology.

For the purposes of this study a surgical procedure is defined as “any medical procedure involving incision with instruments” – this includes major and minor surgery, surgery as a day patient or an inpatient, and surgery with or without anaesthetic. (Caesarean sections are included).

A smartphone is a mobile phone that performs many of the functions of a computer, typically having a touchscreen interface, internet access, and the ability to run apps (e.g. iPhone, Samsung Galaxy, Nexus, HTC, LG and others).

There are no right or wrong answers here – we are simply trying to capture the profile of our partners in the current study – and having variation is a good thing. For instance, if you do not have a smartphone this is not a problem, in fact we are very interested in understanding alternative ways of communicating the information in the My Surgery app.

**Please answer the following questions regarding your previous surgical experience:**

1. How many previous surgical procedures have you had (if none, state zero) _________________

- If your answer to the above is zero, are you due to have upcoming surgery? (circle) YES / NO
- If you have had previous surgery or are due to have surgery, please list the type of surgery you have had or will be having: ____________________________________________________________________________________________________________________________________________________________________________________________________________________________________________________________________________________________________________________________________________________________________________________________

1. If you have had previous surgery, how many years ago was your most recent surgical procedure?

| Less than 1 year ago |
| --- |
| 1-2 years ago |
| 3-5 years ago |
| 5-10 years ago |
| More than 10 years ago  N/A |

1. Have you ever experienced an error in your care in hospital? (circle) YES / NO

- If yes, what happened? ________________________________________________________________________________________________________________________________________________________________________________________________________________________________________________________________________________________________________________

**Please answer the following questions regarding your use of smartphone technology:**

1. Do you own a smartphone? (see definition above) (circle) YES / NO

- If yes, is this an Apple iPhone? (circle) YES / NO
- If yes, are you familiar with using smartphone apps? (e.g. photo apps, news apps, social media apps, maps, weather apps, etc) (circle) YES / NO

1. Do you own or have access to an Apple iPad? (circle) YES / NO

- If yes, are you familiar with using apps on your iPad? (circle) YES / NO

1. Would you use a smartphone app for health-related purposes? (circle) YES / NO
2. Have you downloaded or had a look at the MySurgery app? (circle) YES / NO

If there is anything further you would like to mention in relation to the questions in this questionnaire please feel free to do so below:

**Thanks for your help in responding to these questions**
