# Supplementary material for: A Smartphone App Designed to Empower Patients to Contribute Toward Safer Surgical Care: Qualitative Evaluation of Diverse Public and Patient Perceptions Using Focus Groups
Source: JMIR Mhealth Uhealth. 2021 Apr 8;9(4):e24065. doi: 10.2196/24065 (PMC8063097; doi:10.2196/24065)
Supplement: Multimedia Appendix 5 [file mhealth_v9i4e24065_app5.docx]

**Qualitative thematic analysis: Illustrative quotes**

| **THEME** | **ILLUSTRATIVE QUOTES** |
| --- | --- |
| Perceptions regarding patient involvement in safety | “I think the reason we should be involved in safety is because we’re the people who stand to lose the most when things go wrong”  “Well, I think there’s a kind of responsibility on patients to actually start, it’s about taking responsibility for your care and we need to be moving in that direction and not just leaving it to the various clinicians”  “Nothing about us without us.” So if it’s gonna affect the patient then why aren’t we involved in that”  “In any case if you improve safety the outcomes are better so it’s win win for patients, win win for clinicians and win win for the exchequer”  “Patients can get involved in patient safety but they need encouragement by clinicians and to be told in what ways they can contribute”  “There’s this kind of imbalance of power but at the same time I’m an expert in my own health and sometimes that’s been overlooked because they’re the expert. And so it’s not about kind of diminishing or disparaging their medical expertise it’s about empowering me as a patient and helping me make myself feel safe”  “If you don’t actually know the procedure or what to expect how can the patient then be able to help if there’s not a criteria, or checklist for this kind of thing”  “Can I just say one sort of slight reservation, I mean, I think we should be involved as well for all those reasons that everyone else has said but I suppose one reservation might be that it might start to erode the trust you have in the clinician”  “I think it’s down to, sort of, confidence and assertiveness really and, you know, some people have those kinds of skills and perhaps had practice with that in the workplace, but not everybody”  “What if you've got a patient who actively just wants to be told what to do, doesn’t want to have that responsibility” |
| MySurgery App: concept, content and usability | “I think they ought to know what they can do and not do and if they know they can ask loads of questions and it’s a two-way communication. That’s what this app seems to do, it shows people the possibilities, shows patients the possibilities”  “It’s a good step towards patient empowerment, that’s how I feel about it. Anything that’s going to empower patients can only be a benefit”  “I think the app was brilliant because honestly, it taught me things that I wouldn’t have any idea and I’m thinking now I’m better prepared, because just by reading it doesn’t mean that I’m going into it every day, but things start and I think, oh I didn’t even know I had to do this”  “I liked it, I thought it was very user friendly, it wasn’t information overload so when you opened it and thought, oh god, I’m going to have to sit and spend ages reading through loads of... but it's quite interactive and it’s short”  “You get to the end and you get to the tick and like, yes, I’ve done it... I thought the animations are good, that like breaks up the text a bit”  “Everyone who is going to use it has to have an understanding on how the whole health service works because I think these kinds of discussions we’ve been having the fundamental thing it’s for people to be able to understand that our healthcare is more of, like, a partnership between the patient and the clinicians and for that partnership to work there has to be some trust”  “The information is clean and clear. And I think most people irrespective of age and I’m looking at it from a prism of perhaps learning disabilities could understand it so I think that’s great”  “It just empowers you in a way because it doesn’t mean you have the confidence to say, have you washed your hands, but maybe next time you’ll be able to or maybe you can attempt to, or know that that’s important, or discuss it in a different way with the health worker”  “You shouldn’t have to go through it in a set order, this was annoying as I had to tap tap through until I got to the bit that was relevant to me”  When I stopped and went back later it took me back to the beginning and you have to go through all of the other ones again to get back to where you were so there wasn’t something that saved your progress to date  “You could make it more personal, with a calendar and contacts, or medication reminders, that kind of thing”  “How about links to information about the procedure you are having, or some where to find more information?” |
| Accessibility | “it would need adapting for people that don’t speak English, people who don’t have an iPhone. Blind people, yeah”  “I think that the app is very clear and the language is very clear however there might be groups of society who have a lot of surgery that find it difficult., I’m talking about learning disabilities, Alzheimer’s and so on…, or people that have had neurological trauma and also people who might already be very unwell in hospital”  “My father who was going blind who had severe dementia, who had never seen a computer before really with the best will in the he wouldn’t want it, he wouldn’t use it he’d be scared by it. So I don’t think you’d want to make it accessible for absolutely everyone”  “Don’t make assumptions about the elderly.. cause there is a presumption that the elderly don’t like technology. I’ve spoken to some people in their 70s, I never say to them, do you know how to use an iPad, you just hand it over”  “The difficulty with people in institutions like prison is they I don’t have access to phones so you would need a paper-version of it”  “How about easy-read versions, or incorporating audio, or more visuals. A paper-based version for the techno-phobes amongst us?”  “You cannot with one thing reach all people. You probably won’t be able to reach a certain percentage, but the point is it’s actually much more efficient for most” |
| Implementation | “I think it would be helpful to be using it before your pre-op appointment because that’s probably the longest time you get one to one with a clinician, so yeah, when you get your letter like saying you have a pre-assessment such and such a date, have the information about this app in that letter.  “The letter that goes out to you says, you might want to look at this app first of all and if you have any worries about patient safety, bring them up in the interview, in the consultation. That is embedded in part of the consultation process and accepted”  “It could run in the GPs surgery and whatever, just to raise consciousness and awareness and then people will start asking the questions from that”  “It’s not just the outpatient clinic, it’s not just leaflets, it’s not just through posters, it’s everything, it’s the whole package”  “There’s a dual process going on here as well as education of patients and empowering them, it’s also the education of the clinicians and I think it’s imperative that you speak to clinicians about it”  “So the onus isn’t on you to have to bring it up, like, it’s part of the procedure and it’s a suggested part of the procedure to look at the app before you’ve had the consultation so that you know that question’s coming and that you should prepare and think about it in advance so you can ask the consultant, something like that”  “I suppose if your GP says download that app tonight, the odds of you downloading it might be a bit higher”  “What might be useful would be if the surgical team knew that I had it so that they don’t think “Oh this is a really stroppy patient who’s got all these questions and they’re telling me how to do my job”  “Involve PPGs, Patient Participation Groups and certainly in my area they have digital champions who are there to help people who don’t understand technology”  “so if there’s some sort of linkage that if you link a date so you get a reminder to check the app or something at a given time later on because otherwise it’s very easy to check the list and all that’s on it”  “If you want some help to access this app or you can discuss the information you could contact your local A, B, C...” |
| Untoward impacts | “One danger this is supposed to be supplementary to what the consultants tell us for the leaflets and in writing we’re given, I’m not sure everybody will look at it that way”  “Stress on the doctor patient relationship” |
