# Supplementary material for: A Smartphone App Designed to Empower Patients to Contribute Toward Safer Surgical Care: Qualitative Evaluation of Diverse Public and Patient Perceptions Using Focus Groups
Source: JMIR Mhealth Uhealth. 2021 Apr 8;9(4):e24065. doi: 10.2196/24065 (PMC8063097; doi:10.2196/24065)
Supplement: Supplementary file 5 [file mhealth_v9i4e24065_app4.doc]

Mobilising Knowledge to Patients about Risk in Surgery: a Smartphone Intervention

Chief Investigator and Session Facilitator: Dr Stephanie Russ, stephanie.russ@kcl.ac.uk

Attendees: ______ patient/public participants with experience of surgery (personal or otherwise)

Date and time:

Overall Objectives for the Focus Group:

1. To understand perceptions around the concept of service-users playing an active role in improving safety in healthcare
2. To gather views regarding the perceived feasibility of using smartphone apps as a device for communicating safety information to patients and their carers,
3. To understand views towards MySurgery, particularly in relation to usability, functionality, and design.
4. To understand perceived potential influences of using such apps on the doctor/patient relationship
5. To understand views regarding potential cultural and social influences on the acceptability and use of MySurgery and other such apps– particularly to understand the relevance of the app for minority groups.
6. To understand service user views on how apps such as MySurgery might be integrated into care pathways.

Introduction (instructions for facilitator) (10 mins):

1. Welcome

   Introduce yourself and background, and send the consent form and demographic questions around to the group (re-establish whether it's ok to use tape-recorder).

   Review with the group the following:

   - Thank everyone for attending
   - The broad objectives of the focus group
   - Why they have been asked to participate
   - What will be done with the information collected

2. Explanation of the process

   Ask the group if anyone has participated in a focus group before. Explain that focus groups are being used more and more often in health and human services research.

   About focus groups

   - We learn from you (positive and negative)
   - No right or wrong answers
   - Everyone should be given the opportunity to participate.
   - Don't need permission to speak, can respond directly to others
   - Not trying to achieve consensus, we're gathering information – it's an exploratory process
   - My role: To ask questions, to keep to time, to provide context, to keep on track

Logistics

- Focus group will last about 90 mins
- Feel free to move around
- Where is the bathroom? Fire exit?
- Help yourself to refreshments at any time
- The use of the audio-recorder – only one person talking at a time

3. Ground Rules

   Ask the group to suggest some ground rules to encourage inclusion. After the brainstorm, make sure the following are on the list.

   - First name basis
   - You don't need to agree with others but should respect eachother's views – variation is what we want
   - Information provided in the focus group must be kept confidential
   - Stay with the group and please don't have side conversations
   - Turn off mobile phones if possible
   - Have fun

4. **Turn on Tape Recorder**

5. Ask the group if there are any questions before we get started, and address those questions.

6. Introductions
   - Go around table and ask participants to share a bit about themselves and their experience of surgery

Discussion begins, make sure to give people time to think before answering the questions and don't move too quickly. Use the probes to make sure that all issues are addressed, but move on when you feel you are starting to hear repetitive information.

1. Views regarding patient/carer involvement in safety (approx. 10 mins)

- Do you think that patients *should* play a role in improving safety in hospital settings?

- Do you think that patients and their families can play a role in improving safety in hospital settings?
  PROBE => If so what kind of role? If not, why not?

- Do you think it's easy for patients to play an active role in discussions and decisions around their care?
  PROBE=> What might make this difficult?

- Do you have any experience or personal examples of having influenced the safety of your own or someone else's care?

- Do patients need more education around the kind of role they can play?

- What kind of information do you think patients want to know about safety and the role they can play?
  PROBE => Do they want to know about error rates in their area? Do they want to know what specific actions they can take to improve safety?

- How should this education be delivered?

Introduce the app – acknowledge that not everyone can use it and will come on to that

2. Views regarding MySurgery (15 mins)

- What are your initial impressions of the app?

- Does it seem to contain useful information?
  PROBE=> is the information the right information?

- Do you think the information is actionable?

- Does it seem user friendly?
  PROBE => What works well and what doesn't?

- What changes could be made?
  PROBE=> What other functions could be built in?

- Would you like to use it?

- Is it confusing to have extra info, is It useful to have additional info

3. Use of MySurgery and perceived impact on patient/doctor relationship (10 mins)

- Do you think it would be easy/possible to use the app if you are a surgical patient?
  PROBE=> What would make it harder/easier

- Do you think carers/family could use the app?

- What impact do you think using the app might have on the doctor/patient relationship?

- Do you think doctors would be positive or negative about patients using the app?

- What are the problems with using the app?
  PROBE=> What might be the unintended consequences of using the app?

4. Socio-cultural influences on use and attitudes towards the app (15 mins)

- Will the app be acceptable to all?
  PROBE=> For whom will the app be less useful for? What about people who do not own/use a smartphone/iPad?

- How might we get around the issues identified?

- Can you think of alternative ways of delivering this information?

- Should this impact on the decision to take the app forward?

5. **Implementation of MySurgery and integration into care pathways (10 mins) – moved up**

   - How might the app be integrated into care pathways?
     PROBE=> Should it be recommended by clinicians for example at pre-operative clinics?

   - How else might the app be advertised to service users?

   - For whom will the app be most useful?

6. **How to test the app with real patients?**

7. **Concluding question (5 mins)**

   - Of all the things we discussed, what to you is the most important?

8. **Summary – provide a brief oral summary**

   - Is this an adequate summary? Has anything been missed?

Conclusion

- Thank you for participating. This has been a very successful discussion
- Your opinions will be a valuable asset to the study
- We hope you have found the discussion interesting
- Is there is anything you are unhappy with or wish to complain about
- I would like to remind you that any comments featuring in any reports will be anonymous
- Before you leave, please hand in your completed personal details questionnaire
